# Neurofilament light chain is a promising serum biomarker in spinocerebellar ataxia type 3

**DOI:** 10.1186/s13024-019-0338-0

**Published:** 2019-11-04

**Authors:** Quan-Fu Li, Yi Dong, Lu Yang, Juan-Juan Xie, Yin Ma, Yi-Chu Du, Hao-Ling Cheng, Wang Ni, Zhi-Ying Wu

**Affiliations:** 1 Department of Neurology and Research Center of Neurology in Second Affiliated Hospital, and Key Laboratory of Medical Neurobiology of Zhejiang Province, Zhejiang University School of Medicine, 88 Jiefang Road, Hangzhou, 310009 China; 2 Department of Neurology and Institute of Neurology, First Affiliated Hospital, Fujian Medical University, Fuzhou, China

**Keywords:** Neurofilament light chain, Spinocerebellar ataxia type 3, Serum biomarker

## Abstract

**Background:**

Spinocerebellar ataxia type 3 (SCA3) is the most common subtype of autosomal dominantly inherited spinocerebellar ataxias (SCAs). No validated blood biomarker is available to assess either disease progression or therapeutic response. Neurofilament light chain (NfL) was recently proposed as a serum biomarker for many neurodegenerative disorders. The present study investigated whether NfL was a promising serum biomarker for SCA3.

**Methods:**

Seventeen SCA3 patients and 9 controls were enrolled in cohort A, and 116 SCA3 individuals (preclinical and patients) and 91 controls were recruited as cohort B. We assessed whether serum NfL correlated with cerebrospinal fluid (CSF) NfL in cohort A and correlations between serum NfL levels and clinical features and brain volumes were determined in cohort B. The single-molecule array method was used to measure serum NfL levels. Disease severity was determined using the scale for the assessment and rating of ataxia (SARA) and the international cooperative ataxia rating scale (ICARS). Cerebellar and brainstem volumes were assessed using MRI neuroimaging measurements.

**Results:**

Serum/CSF NfL levels in cohort A were elevated in SCA3 patients, and serum and CSF NfL exhibited a significant positive correlation (*r* = 0.9179, *p* < 0.0001). Levels of serum NfL in cohort B were significantly higher in preclinical SCA3 (15.03 ± 7.49 vs 6.88 ± 2.72 pg/ mL, *p* < 0.0001) and manifest SCA3 subjects (37.56 ± 13.47 vs 9.07 ± 6.02 pg/ mL, *p* < 0.0001) compared to those in controls. Serum NfL concentrations increased from early disease stage to the next stage. Levels of serum NfL in *ATXN3* mutation carriers were positively associated with SARA (*r* = 0.5458, *p* < 0.0001) and ICARS scores (*r* = 0.5522, *p* < 0.0001). Significant negative associations with cerebellar volumes (*r* = − 0.4217, *p* = 0.0003) and brainstem volumes (*r* = − 0.4263, *p* = 0.0003) were observed. All changes remained significant after adjustment for age and CAG repeat.

**Conclusions:**

Levels of serum NfL were significantly elevated in SCA3 individuals and correlated with disease severity. Serum NfL is a promising serum biomarker of disease onset and progression, and a potential candidate biomarker of treatment response in SCA3.

## Background

Spinocerebellar ataxias (SCAs) are a worldwide clinically and genetically heterogeneous group of autosomal dominant neurodegenerative disorders, and there are more than 40 different genetic subtypes. Among these dynamic repeat expansion diseases, SCA1, SCA2, SCA3, SCA6, SCA7 and SCA17 share the same pathogenic mechanism of CAG trinucleotide repeat expansions encoding elongated polyglutamine tracts. SCA3 is one of the most common subtypes across geographical regions and ethnic populations, especially in Portugal [1], Brazil [2] and China [3]. SCA3 is a slowly progressing neurological disease. The high degree of clinical variability shows five distinct subtypes of SCA3 [4, 5]. Despite the availability of clinical rating scales currently used in clinical practice, such as the scale for the assessment and rating of ataxia (SARA) and international cooperative ataxia rating scale (ICARS), these scales exhibit low sensitivity for slowly progressing diseases, such as SCA3, and are of limited use in clinical trials [6]. Therefore, a reliable molecular biomarker is urgently needed to monitor SCA3 disease and assess treatment outcomes, especially for future clinical interventional trials.

Significant effort in the past few years was devoted to the search for biomarkers of SCAs. Transcriptional biomarkers were reported in a cross-sectional study with SCA3 patients and controls, and a pool of upregulated genes may be biomarkers for SCA3 [7]. Peripheral glutathione peroxidase activity was a promising oxidative stress disease biomarker in SCA3 [8]. Several candidate biofluid biomarkers were proposed for SCAs, including serum cytokines [9], CSF tau [10], and micro-RNA [11]. Neuroimaging indicators, such as magnetic resonance imaging (MRI) and magnetic resonance spectroscopy (MRS), demonstrated good sensitivity in patients with SCAs and premanifest carriers of SCA causative mutations [12–14]. However, none of these biomarkers are widely accepted to fully track SCA3 from its preclinical phase to the disease stage. Several limitations remain for current candidate biomarkers. Biofluid biomarkers have not proven useful because of insufficient sensitivity and specificity. Imaging biomarkers are generally expensive and potentially difficult to access.

Neurofilaments are the major elements of cytoskeletal proteins in neurons and are composed of three subunits called neurofilament light chain (NfL), medium chain and heavy chain [15]. NfL is detected in the CSF and plasma/serum after damage to nerves. Several recent studies reported NfL as a promising marker in neurodegenerative diseases, such as Huntington’s disease (HD) [16], Alzheimer’s disease [17], amyotrophic lateral sclerosis [18], multiple sclerosis [19], and frontotemporal dementia [20], and peripheral nerve disorders, such as Charcot-Marie-Tooth disease [21]. A meta-analysis supports the use of NfL as a biomarker of neuroaxonal damage [22]. A small cohort pilot study revealed that serum NfL levels were increased in repeat-expansion SCAs and distinguished SCAs from sporadic adult-onset ataxia [23]. However, only 20 SCAs patients were included and the correlation between NfL level and disease severity was not analyzed. Whether NfL may be used as a biomarker is not known, and NfL concentrations must be examined in a large cohort of SCA3 patients.

Therefore, we determined the correlation of NfL levels in the CSF and serum. We also performed a prospective study to assess NfL as a disease biomarker in SCA3 and analyzed the changes in NfL at different stages of SCA3.

## Methods

### Participants

A total of 133 SCA3 individuals from 113 families were recruited from the Second Affiliated Hospital of Zhejiang University School of Medicine between March 30, 2015 and July 12, 2018. The 133 SCA3 individuals were molecularly diagnosed as previously reported [3] at the Research Center of Neurology in Second Affiliated Hospital. A total of 100 phenotypically normal individuals with negative genetic screening for *ATXN3* were selected as controls, including 52 spouses or siblings of SCA3 individuals, and 48 unrelated individuals with no known family history of SCA3. Age and gender were considered in control selection.

### Study design, clinical scales and neuroimaging assessments

Demographic data, clinical characteristics, brain MRI and blood/CSF samples were collected from the participants. Age was defined as age at sample collection. Twenty-six participants, including 17 SCA3 patients and 9 controls, were enrolled in Cohort A to assess the correlation between serum and CSF NfL concentrations. A total of 207 participants, including 116 *ATXN3* mutation carriers (26 preclinical and 90 manifest SCA3) and 91 controls, were enrolled as an independent Cohort B for further research. Controls in Cohort B were divided into control 1 for preclinical and control 2 for manifest SCA3.

Clinical disease severity using SARA and ICARS scales was recorded in all *ATXN3* mutation carriers. *ATXN3* mutation carriers in Cohort B were classified into two groups based on SARA (score 0~40) as preclinical (SARA score < 3) and manifest subjects (SARA score ≥ 3) [24]. Manifest SCA3 subjects were further separated into two subgroups according to the median SARA score: Stage 1 (SARA score 3~11) and Stage 2 (SARA score ≥ 11). Preclinical SCA3 subjects were further separated into two subgroups of early preclinical and late preclinical using the median predicted number of years to onset of manifest disease (7.6 years) as the threshold.

To measure the volumes of the cerebellum and brainstem, 68 *ATXN3* mutation carriers underwent whole-brain T1-weighted images, including 66 manifest and 2 preclinical SCA3 subjects. SPM 8 software (http://www.fil.ion.ucl.ac.uk/spm) on MATLAB 2018a was used to perform brain segmentation. We obtained the volumes of gray matter, white matter and CSF filling areas from each subject, which were used to calculate the total intracranial volumes (TIV). The volumes of brainstem and cerebellum were manually measured using ITK-SNAP software (Version 3.6.0) [25]. In the correlation analyses between serum NfL concentrations and the volumes of brainstem and cerebellum, the TIV was regressed out as a covariate to eliminate the influence of TIV.

### Serum/CSF NfL measurement

For each participant, venous blood was collected in BD Vacutainer tubes (Plymouth PL6 7BP, UK) containing separation gel. Blood samples were rested for 30 min at room temperature and centrifuged at 2000×g for 10 min at 4 °C to obtain serum. CSF samples were collected via lumbar puncture at the L3/4 or L4/5 space. The first 1 mL of CSF was discarded, and the next 5–10 mL of CSF was collected. CSF samples were processed in a maximum interval of 10 min after collection. White blood cell and erythrocyte counts were determined. CSF samples were processed on ice and centrifuged at 400×g for 10 min at 4 °C to remove cells. Serum and CSF samples were frozen and stored in Protein LoBind Tubes (Eppendorf AG, Germany) at − 80 °C. Samples were shipped frozen on dry ice for analyses. NfL was quantified using the single-molecule (Simoa) array method [20] and the Simoa NF-light assay (Quanterix, MA, US) on an HD-1 platform (GBIO, Hangzhou, China). Each plate contained calibrators and quality controls. All NfL values were within the linear ranges of the assays. The inter- and intra-assay coefficients of variation were below 10%. Serum samples were diluted at a ratio of 1:4. CSF samples were prediluted at a ratio of 1:100. Operators were unaware of participants’ disease status.

### Statistical analysis

The NfL concentrations in the serum and CSF were nonnormally distributed. Therefore, Mann-Whitney and Kruskal-Wallis tests were used to compare groups. Bonferroni correction was used for multiple comparisons. Correlations between variables were assessed using Spearman correlation coefficient. Receiver operating characteristic (ROC) curve was used for the sensitivity analysis of NfL cutoff values for SCA3. Overall sensitivity and specificity were assessed as areas under the curve (AUC). Because age and CAG repeat count are known prognostic factors for SCA3 progression, we used a polynomial (quadratic) model to describe the association between NfL concentrations, age and CAG repeat count. Because the number of patients was too small, CAG repeats less than 69 and greater than 79 were excluded from the analysis. The two adjacent CAG repeat groups were combined for analysis, such as CAG = 69, 70. The threshold for statistical significance was *p* < 0.05, and the analyses were using GraphPad Prism 7.0 (GraphPad Inc., La Jolla, CA, USA).

## Results

### Demographic features of SCA3 participants and controls

A total of 133 *ATXN3* mutation carriers and 100 healthy controls were enrolled into this study. There were no significant differences in the age or gender of SCA3 and controls (*p* > 0.05). The demographic details of Cohort A and Cohort B are shown in Table 1.

**Table 1:** The demographic features and NfL concentration in Cohort A and B

Group	Cohort A		Cohort B			
	Control	SCA3	Control1	Preclinical SCA3	Control2	Manifest SCA3
n	9	17	21	26	70	90
Age	54.33 ± 11.69	49.88 ± 11.75	31.62 ± 11.61	31.38 ± 9.30	43.13 ± 12.66	43.90 ± 12.16
Sex (M/F)	4/5	6/11	8/13	9/17	31/39	41/49
Expanded CAG repeats	N/A	73.18 ± 3.64	N/A	72.62 ± 4.97	N/A	73.91 ± 4.04
SARA	N/A	13.82 ± 5.56	N/A	0.73 ± 0.83	N/A	11.72 ± 6.02
ICARS	N/A	35.00 ± 11.06	N/A	1.81 ± 1.81	N/A	29.04 ± 13.90
Disease duration (ys)	N/A	5.82 ± 3.56	N/A	N/A	N/A	6.59 ± 5.53
Total intracranial volume (mL)	N/A	N/A	N/A	1416.00 ± 128.60 (*n* = 2)	N/A	1496.00 ± 231.20 (*n* = 66)
Cerebellum (mL)	N/A	N/A	N/A	148.17 ± 87.77 (*n* = 2)	N/A	127.07 ± 21.27 (*n* = 66)
Brainstem (mL)	N/A	N/A	N/A	22.17 ± 1.38 (*n* = 2)	N/A	20.05 ± 4.66 (*n* = 66)
Serum NfL (pg/mL)	9.10 ± 3.29	41.50 ± 14.78	6.88 ± 2.72	15.03 ± 7.49	9.07 ± 6.02	37.56 ± 13.47
CSF NfL (pg/mL)	471.70 ± 210.40	4262.00 ± 1762.00	N/A	N/A	N/A	N/A

Values are mean ± SD. *SARA* Scale for the assessment and rating of ataxia, *ICARS* International cooperative ataxia rating scale, *N/A* Not applicable

### Serum NfL correlated with CSF NfL in SCA3

**Fig. 1:**
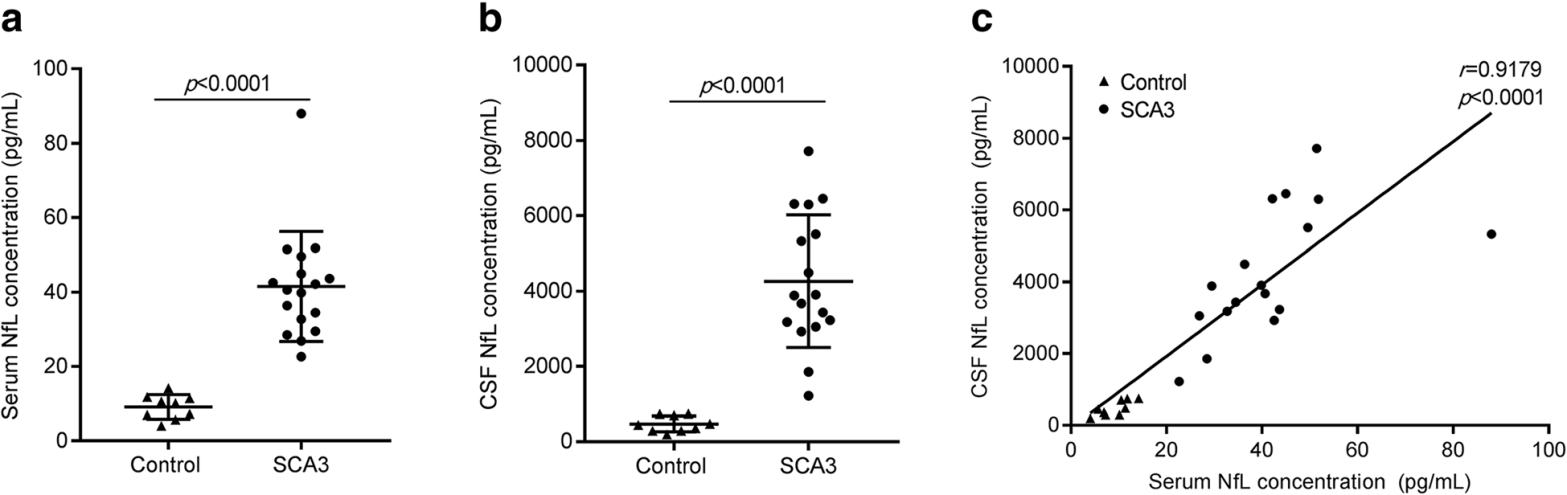
Correlations of NfL level between serum and CSF in cohort A. **a**. CSF NfL concentration is significantly higher in SCA3 patients than controls. **b**. Serum NfL concentration is significantly higher in SCA3 patients than controls. **c**. The correlation of NfL between serum and CSF is significantly positive

To assess the correlation between serum and CSF NfL, an independent Cohort A of 26 participants (17 SCA3 patients and 9 controls) was studied first. The mean concentrations of NfL in the CSF and serum were significantly higher in SCA3 patients than controls (4262.00 ± 1762.00 pg/mL vs 471.70 ± 210.40 pg/mL, *p* < 0.0001; 41.50 ± 14.78 pg/mL vs 9.10 ± 3.29 pg/mL, *p* < 0.0001; Fig. 1a-b, Table 1). The mean CSF NfL concentration was 102 times higher than the serum NfL in SCA3 patients. There was a significant positive correlation between NfL concentrations in the CSF and serum (*r* = 0.9179, *p* < 0.0001, Fig. 1c).

### Serum NfL concentrations were elevated in SCA3

**Fig. 2:**
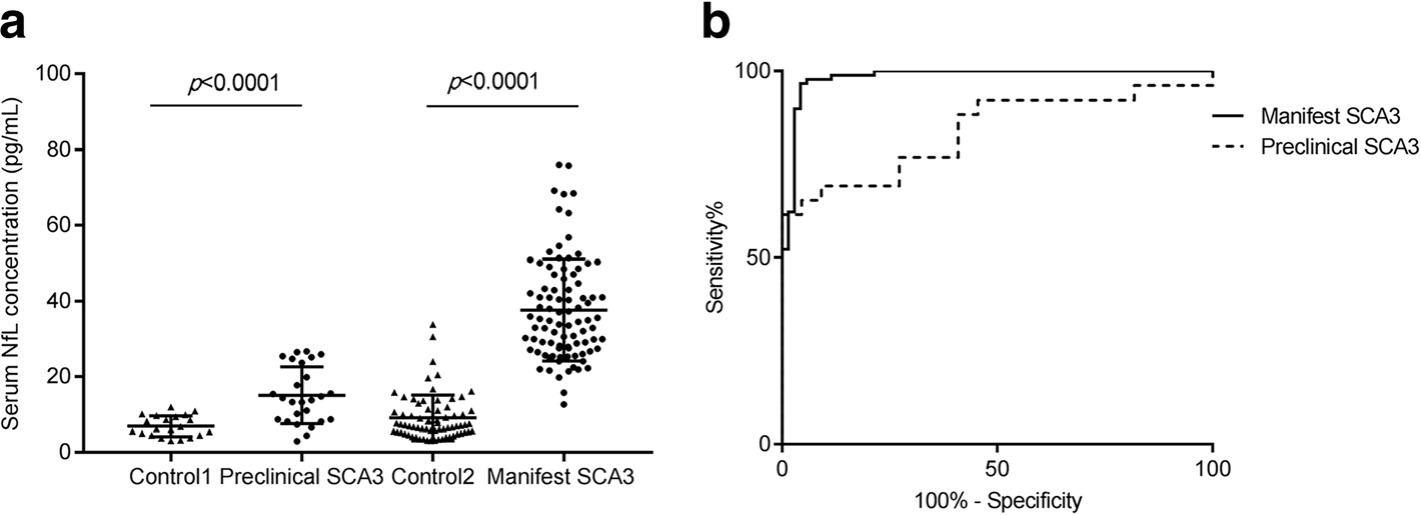
Levels of serum NfL in cohort B. **a**. Serum NfL concentration is significantly increased in preclinical (*n* = 26) and manifest SCA3 subjects (*n* = 90) compared to controls. **b**. Receiver operator curve (ROC) of NfL concentration show good sensitivity and specificity for detecting preclinical SCA3 and manifest SCA3

We studied serum NfL concentrations in a large independent cohort (Cohort B). Levels of serum NfL were significantly higher in preclinical SCA3 (15.03 ± 7.49 vs 6.88 ± 2.72 pg/ mL, *p* < 0.0001) and manifest SCA3 (37.56 ± 13.47 vs 9.07 ± 6.02 pg/ mL, *p* < 0.0001) compared to controls (Table 1 and Fig. 2a). Additionally, ROC showed that serum NfL concentration distinguished *ATXN3* mutation carriers from controls with high accuracy (Fig. 2b). The AUC of manifest SCA3 was 0.984 (95% CI 0.965, 1.002), and AUC for preclinical SCA3 was 0.839 (95% CI 0.724, 0.955). As shown in Additional file 1: Table S1, a concentration of 20 pg/mL as the cutoff value showed a pretty good sensitivity and specificity for detecting manifest SCA3 patients (97% and 94%, respectively), and a concentration of 10 pg/mL as the cutoff value showed decreased sensitivity and specificity for detecting preclinical SCA3 (69% and 91%, respectively).

### Serum NfL concentration correlated with disease severity in SCA3

**Fig. 3:**
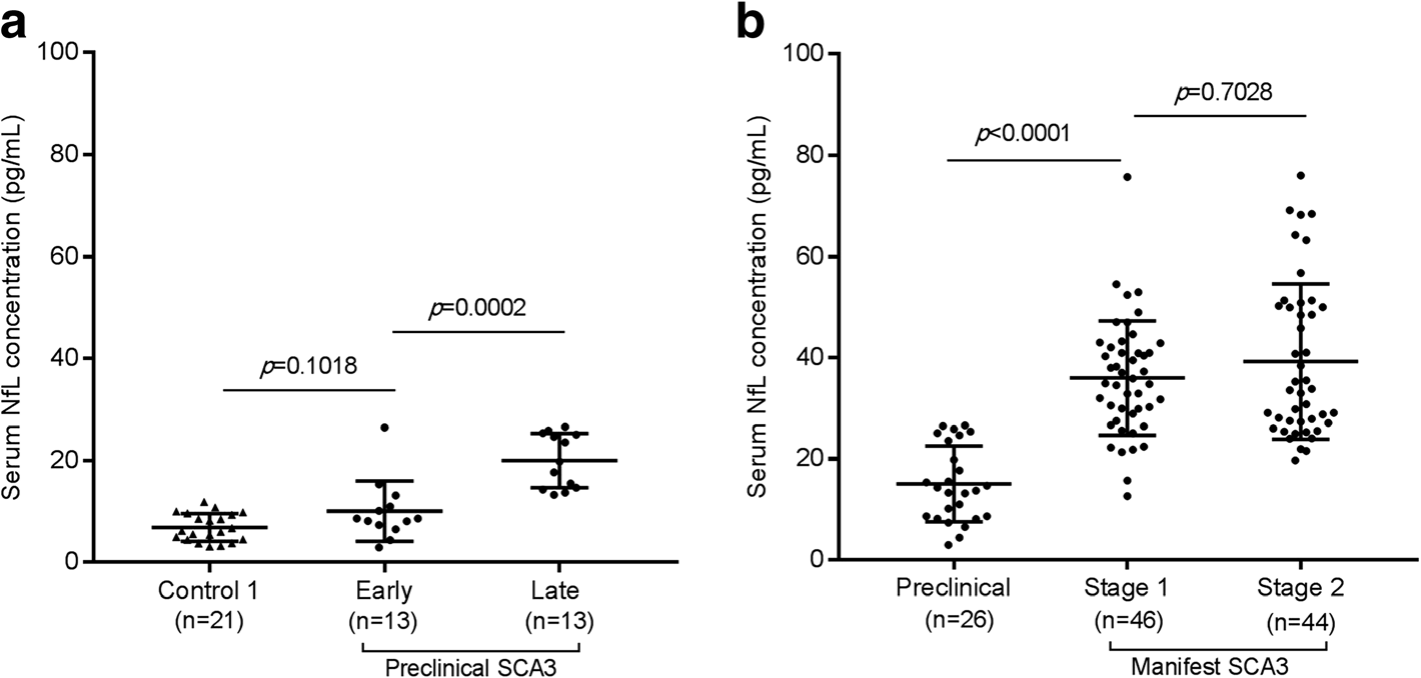
Associations between serum NfL and disease stages of SCA3. **a**. For preclinical SCA3, serum NfL concentration is significantly higher in the late preclinical than the early preclinical SCA3. **b**. Serum NfL concentration is significantly higher in the manifest stage of SCA3 than preclinical SCA3. No significant difference is seen between Stage 1 and Stage 2

Participants were classified into 3 subgroups according to SARA scores including 26 preclinical SCA3 individuals, 46 Stage 1 and 44 Stage 2 SCA3 patients. For preclinical SCA3 individuals, no differences in serum NfL were observed between the early preclinical and controls (*p* = 0.1018, Fig. 3a). However, subjects in late preclinical SCA3 showed higher NfL levels compared to the early preclinical SCA3 (20.01 ± 5.31 vs 10.05 ± 5.92 pg/ mL, *p* = 0.0002, Fig. 3a). Serum NfL concentration was significantly higher in the manifest stage than the preclinical SCA3 (*p* < 0.0001). There was no difference in serum NfL between Stage 1 and Stage 2 in manifest SCA3 patients (Fig. 3b).

**Fig. 4:**
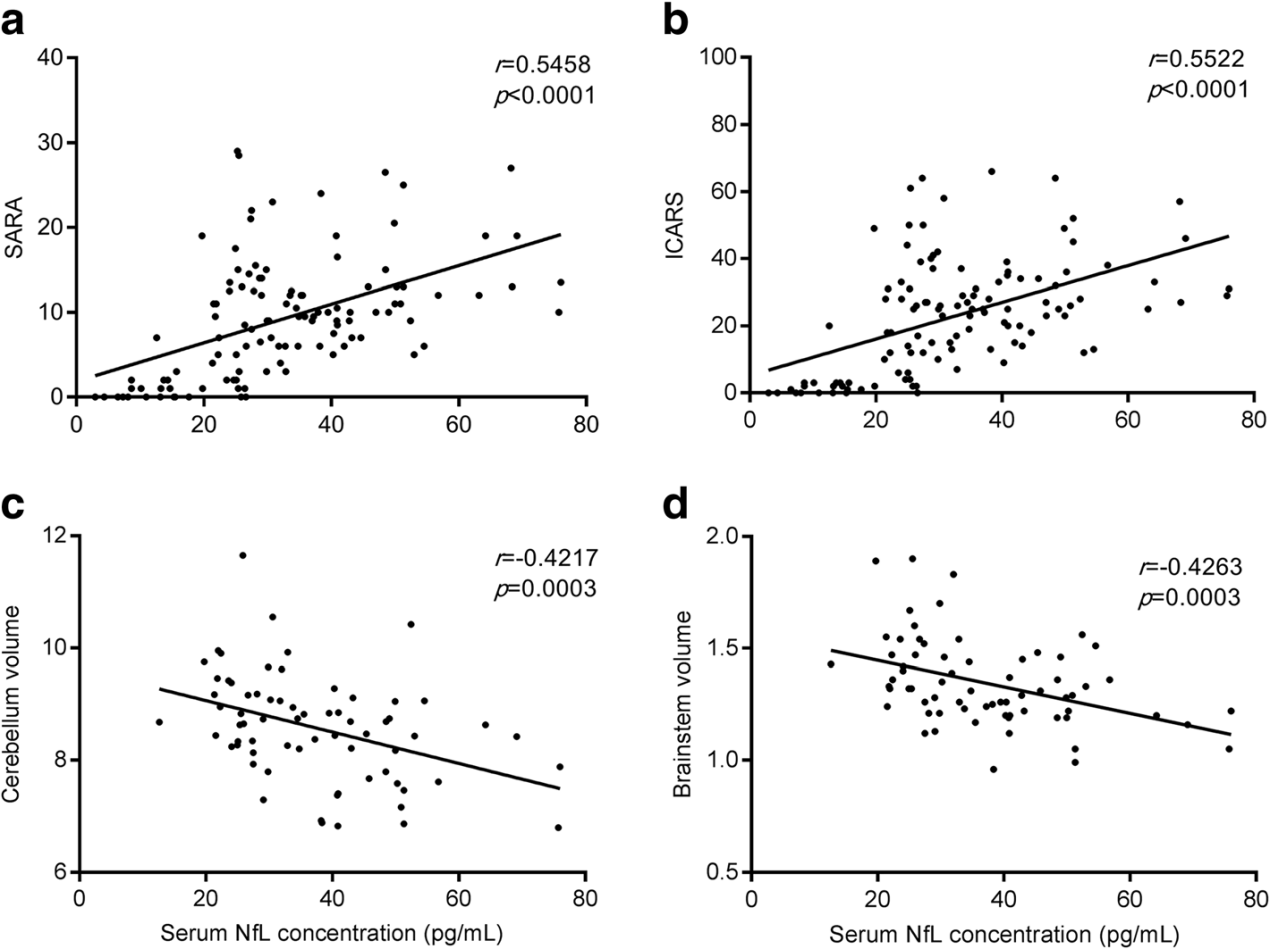
Associations between serum NfL levels and clinical scales, cerebellum and brainstem volumes. **a-b**. Serum NfL concentration positively correlate with SARA and ICARS scores. **c-d**. Serum NfL concentration negatively correlate with cerebellum and brainstem volumes, expressed as percentages of total intracranial volume

Serum NfL concentrations in the *ATXN3* mutation carriers were positively associated with SARA and ICARS scores (*r* = 0.5458, *p* < 0.0001 and *r* = 0.5522, *p* < 0.0001; Fig. 4a-b). Significant negative associations with cerebellar volumes and brainstem volumes were observed (*r* = − 0.4217, *p* = 0.0003 and *r* = − 0.4263, *p* = 0.0003 respectively; Fig. 4c-d). These associations remained significant after adjustment for the effects of age and expansion CAG repeat count (*r* = 0.260, *p* = 0.005 for SARA, *r* = 0.252, *p* = 0.007 for ICARS, *r* = − 0.418, *p* < 0.0001 for cerebellar volumes and *r* = − 0.419, *p* < 0.0001 for brainstem volumes). Higher serum NfL concentration was associated with more serious disease, smaller cerebellar and brainstem volume.

A positive association between serum NfL concentration and age was observed in the controls and SCA3 individuals (Additional file 2: Figure S1). This association was roughly linear in controls. The association was nonlinear in *ATXN3* mutation carriers, and serum NfL concentration increased with increasing CAG repeat count and age. For a given CAG repeat, the steepness of the slope declined with increasing age.

## Discussion

NfL is a neurofilament subunit in the central nervous system and it is particularly abundant in axons. NfL in the plasma was recently reported as a potential prognostic biomarker of disease onset and progression in HD [16]. The polyglutamine (polyQ) SCAs share similar pathogenic mechanism to HD, with CAG trinucleotide repeat expansions underlying both conditions. ATXN3 protein is widely expressed in peripheral and neuronal tissues. ATXN3 is a deubiquitinating enzyme that is regulated via ubiquitination. The precise pathogenic mechanism that is triggered by dynamic mutation within *ATXN3* in SCA3 patients is not known, but emerging evidence shows that expansion of the polyQ affects many properties of the ATXN3 protein, such as stability and degradation, which result in loss and/or gain of function of this protein and lead to cellular dysfunction and neuronal cell death [26, 27].

Levels of serum NfL were measured recently in a small cohort of patients with degenerative ataxias, including 25 patients with multiple system atrophy, 25 patients with sporadic adult-onset ataxia, and 20 patients with SCAs (SCA 1, 2, 3 and 6) [23]. The results revealed increased serum NfL in SCAs compared to controls. However, there are some limitations in this study. First, only 20 SCAs patients (SCA3 *n* = 8) were included, and NfL was measured only in the serum and not in the CSF. Second, a correlation between NfL levels and disease severity was not determined because of the limited number of patients. Third, individuals in the preclinical stage were not enrolled, and MRI imaging parameters were not included.

The current study quantified NfL using an ultrasensitive Simoa method in a relatively large cohort of SCA3 patients, including manifest and preclinical individuals. Our findings showed that serum NfL concentrations exhibited a good correlation with CSF NfL concentrations. Serum NfL levels were also considerably higher in the SCA3 population compared to controls, and increased serum NfL levels were observed in manifest SCA3 subjects and preclinical SCA3 individuals. Serum NfL levels increased with SCA3 disease severity and correlated with clinical scales and neuroimaging markers of SCA3. Our data showed that serum NfL may be a promising molecular biomarker for the SCA3 population.

SARA and ICARS scores are clinical outcomes based neurological examination, and these scales are most commonly used for determinations of disease activity in SCAs patients. Clinical scales are obtained without the need for sophisticated equipment. The advantages of clinical scales are the relatively low requirements of time and cost. However, clinical scales have some subjective questions and may vary between examiners. Clinical scales are also insensitive to subtle changes in a short period of time. The use of current clinical scales is limited in the preclinical stage of SCA3. In contrast, serum NfL is a peripheral blood-based and easily obtained measurement. Therefore, it is a promising biomarker that is worth further study for application in clinical practice.

It remains unclear how early a therapeutic intervention must be administered in SCA3. In fact, a series of nonmotor phenotypes, such as painful muscle cramps, hyperreflexia and sensory abnormalities, is detected before the appearance of ataxia. Pontocerebellar structures may already be reduced in size prior to the onset of symptoms [7, 28]. Mitochondrial DNA common deletions are accumulated in the preclinical stage of SCA3 [29]. Although the pathophysiological process in preclinical SCA3 subjects is not clear, changes were demonstrated in animal models. The transcriptional changes in SCA3 mouse brain occurred prior to the onset of motor and coordination deficits [30]. Citalopram treatment ameliorated motor coordination and attenuated disease progression attenuated in an SCA3 mouse model [31]. These results emphasize the importance of early intervention. Our results showed elevated serum NfL in the late stage of preclinical SCA3 subjects. Although there is currently no effective therapy, the present study demonstrated that serum NfL is a promising peripheral serological indicator to determine the optimal moment for treatment at the preclinical stage of SCA3. Preclinical SCA3 subjects with high NfL levels should receive early treatment.

Our data also showed that serum NfL differentiated the preclinical and early stages (Stage 1) of manifest SCA3, but a reduced sensitivity in the late stage (Stage 2) was observed. Predicted NfL concentrations at older ages became similar in SCA3 patients. For a given CAG repeat, a downward trend of NfL levels was observed with higher SARA scores and increasing age. Similar results were also seen in manifest HD patients [16]. The neuroimaging findings were consistent with this phenomenon. We hypothesize that NfL levels will reach an equilibrium state with neuronal loss in the late stage of neurodegenerative diseases. The NfL concentration becomes slightly elevated and inversely decreases at older ages. Further research is needed to study this mechanism and confirm the results.

However, NfL is not a disease-specific biomarker. Elevated NfL is observed in many neurological disorders, including neurodegenerative diseases, demyelination disease, peripheral neuropathy and traumatic brain injuries [16–23, 32]. However, the specific parts of the nervous system that drive the NfL increase are unknown. The precise releasing process of NfL also remains unclear. NfL reflects the neurodegenerative and neuronal damage processes. Imaging and postmortem studies may provide deeper insight into the underlying pathophysiology of NfL.

## Conclusion

In summary, we identified significantly elevated NfL levels in the SCA3 population. This study provides strong evidence to support serum NfL as a promising blood biomarker of disease onset, stage and severity. Serum NfL may also be a potential candidate biomarker of treatment response in future clinical trials of SCA3. Our study is the first large, comprehensive study of NfL levels focusing exclusively on SCA3 disease. Further studies are needed in other polyQ SCA diseases. However, there are some limitations in this study. First, we only demonstrated the baseline data of SCA3. Longitudinal data of NfL concentrations in the serum and disease progression are currently in progress. Second, not all participants completed the brain MRI scan for personal reasons.

## Supplementary information

**Additional file 1: Table S1.** Sensitivity and specificity of NfL for detecting individuals with SCA3.

**Additional file 2: Figure S1.** Associations between serum NfL concentration and age-CAG repeat count of *ATXN3* in a polynomial model. **A.** The straight line shows the association between serum NfL concentration and age for all controls. **B-F.** The curved lines show the quadratic fit for all participants with a given CAG repeat count. As the number of CAG repeats increase, the level of serum NfL become higher and steeper.

## Data Availability

The datasets used and/or analyzed in the current study are available from the corresponding author on reasonable request.

## Abbreviations

HD: Huntington’s disease
ICARS: International cooperative ataxia rating scale
NfL: Neurofilament light chain
polyQ: polyglutamine
SARA: Scale of assessment and rating of ataxia
SCA3: Spinocerebellar ataxia type 3
Simoa: Single-molecule array
