# Supplementary material for: Neurofilament light chain is a promising serum biomarker in spinocerebellar ataxia type 3
Source: Mol Neurodegener. 2019 Nov 4;14:39. doi: 10.1186/s13024-019-0338-0 (PMC6829913; doi:10.1186/s13024-019-0338-0)
Supplement: Supplementary file 1 — Additional file 1: Table S1. Sensitivity and specificity of NfL for detecting individuals with SCA3. [file 13024_2019_338_MOESM1_ESM.docx]

**Table S1. Sensitivity and specificity of NfL for detecting individuals with SCA3**

|  | **Preclinical SCA3** | |  | **Manifest SCA3** | |
| --- | --- | --- | --- | --- | --- |
| **NfL (pg/mL)** | **Sensitivity (%)** | **Specificity (%)** |  | **Sensitivity (%)** | **Specificity (%)** |
| >5 | 92 | 31 |  | 100 | 23 |
| >10 | 69 | 91 |  | 100 | 70 |
| >15 | 42 | 100 |  | 99 | 88 |
| >20 | 27 | 100 |  | 97 | 94 |
| >25 | 15 | 100 |  | 87 | 97 |
| >30 | N/A | N/A |  | 63 | 97 |

N/A= Not applicable.
