# Supplementary figures and images for: Neurofilament light chain is a promising serum biomarker in spinocerebellar ataxia type 3
Source: Mol Neurodegener. 2019 Nov 4;14:39. doi: 10.1186/s13024-019-0338-0 (PMC6829913; doi:10.1186/s13024-019-0338-0)

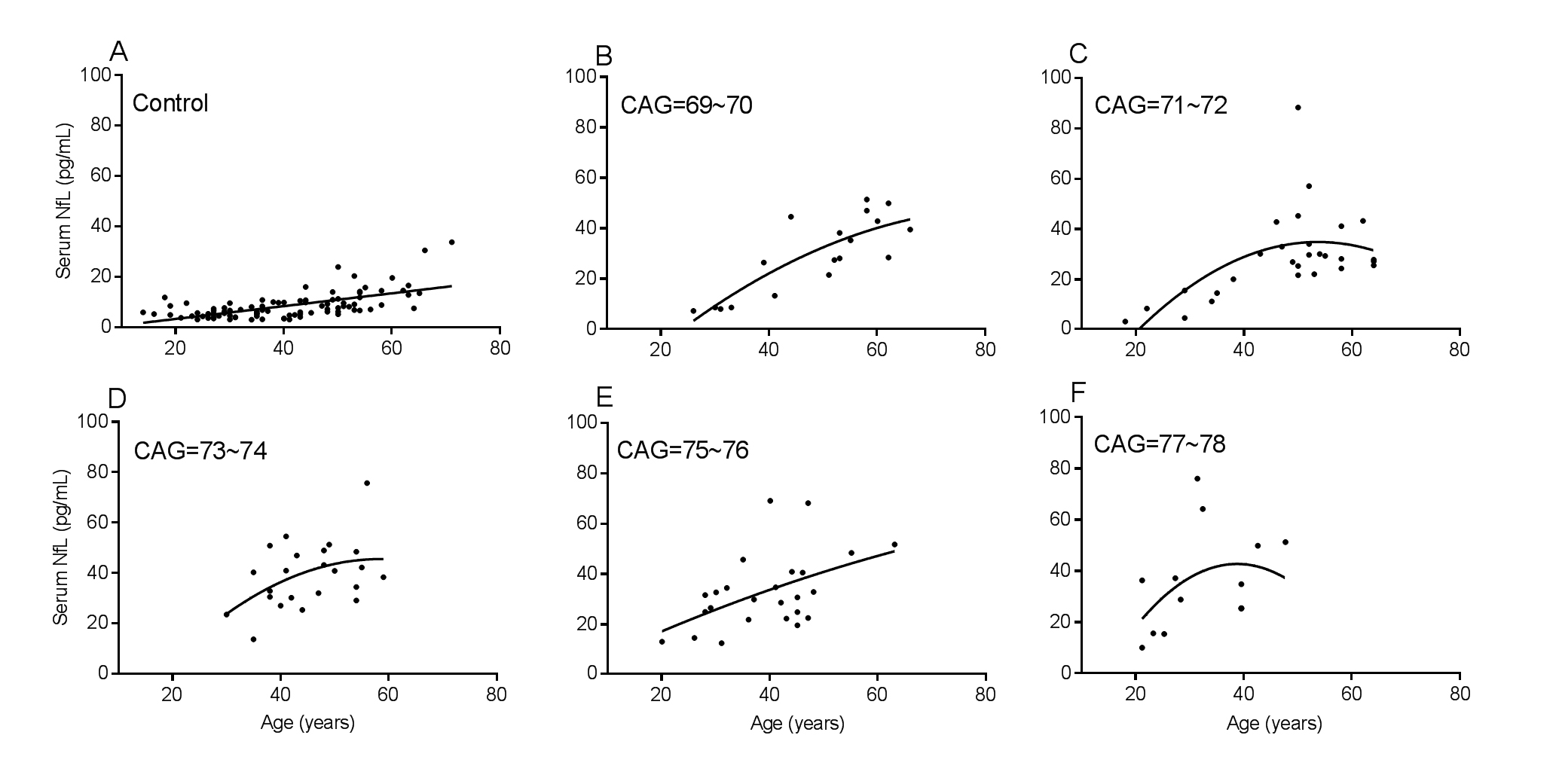

Supplement: Supplementary file 2 — Additional file 2: Figure S1. Associations between serum NfL concentration and age-CAG repeat count of ATXN3 in a polynomial model. A. The straight line shows the association between serum NfL concentration and age for all controls. B-F. The curved lines show the quadratic fit for all participants with a given CAG repeat count. As the number of CAG repeats increase, the level of serum NfL become higher and steeper. [file 13024_2019_338_MOESM2_ESM.jpg]
